# DNAzyme-Based Target-Triggered Rolling-Circle Amplification for High Sensitivity Detection of microRNAs

**DOI:** 10.3390/s20072017

**Published:** 2020-04-03

**Authors:** Chen Liu, Jialun Han, Lujian Zhou, Jingjing Zhang, Jie Du

**Affiliations:** State Key Laboratory of Marine Resource Utilization in South China Sea, College of Materials Science and Engineering, Hainan University, Haikou 570228, China; chen__liu@126.com (C.L.); jialun_han@126.com (J.H.); a2735901314@163.com (L.Z.); zhangjingjingaoxue@163.com (J.Z.)

**Keywords:** microRNAs, deoxyribozyme, rolling-circle amplification, signal amplification

## Abstract

MicroRNAs regulate and control the growth and development of cells and can play the role of oncogenes and tumor suppressor genes, which are involved in the occurrence and development of cancers. In this study, DNA fragments obtained by target-induced rolling-circle amplification were constructed to complement with self-cleaving deoxyribozyme (DNAzyme) and release fluorescence biomolecules. This sensing approach can affect multiple signal amplification permitting fluorescence detection of microRNAs at the pmol L^−1^ level hence affording a simple, highly sensitive, and selective low cost detection platform.

## 1. Introduction

MicroRNAs are endogenous small RNAs that regulate genes, almost all of which are associated with known physiological or pathological processes, thus can reflect a person’s health status [1]. Furthermore, certain microRNAs are involved in the formation and death of tumor cells, and the expression of microRNAs changes during these processes [2], thus cancer may be detected, treated, and also prevented by studying such changes. Biological microRNAs may serve as a drug or as a diagnostic tool in disease treatment [3,4]. In addition to oncology disease, there are many other conditions associated with microRNAs, including diabetes, Parkinson’s disease, cardiovascular disease [5], HIV, and nervous system conditions [6]. In these diseases, it is equally important to monitor the expression of microRNAs [7].

MicroRNAs are characterized by short sequences, high similarity, and low quantity of expression. Traditional detection methods include mainly real-time quantitative PCR using the microarray method and the RNA imprinting method. However, these methods are not sufficiently sensitive, the detection speed is slow, the equipment is expensive, and operational requirements are demanding [8]. In order to improve these deficiencies, we chose DNA fluorescent biosensor as the research foundation. Rolling circle amplification (RCA) and deoxyribozyme (DNAzyme) were combined to conceive a detection platform with high sensitivity and high selectivity. 

Fluorescence measurement is attractive because of its high sensitivity, simple operation, and low cost, and because it is widely accepted for biological monitoring purposes [9]. Using reactive dyes as specific probes, Jiang et al. were able to monitor the rolling ring amplification reaction in real time [10]. Wan et al. used a target-assisted self-cleavage of fluorescently modified deoxyribozyme to develop a sensor that can detect different kinds of microRNAs and at the same time amplify the fluorescence signal [11]. Using the self-RCA technology, Chen et al. designed a biosensor for fluorescent signal amplification and demonstrated applicability to tumor-related microRNAs, detecting concentrations as low as the fmol L^−1^ level [12]. Zhuang et al. described a novel DNA nanomachine constructed using RCA technology and DNAzyme with fmol L^−1^ level detection capability to measure microRNAs [13]. Using the specific fluorescence response of thioprotein T to g-tetrad formed by RCA products, hong-xin Jiang et al. designed a microRNA detection platform with detection limit of 4 am, which also proved the feasibility of microRNA analysis in human lung cancer cells [14]. Thus, the combination of RCA and DNAzyme for use as a fluorescence detection platform is viable.

Rolling-circle amplification is a novel thermostated nucleic acid amplification technology that can amplify targets as primers and convert deoxyribonucleoside triphosphates (dNTPs) into single-stranded DNA under enzymatic catalysis [15]. The amplified fragment is essentially an infinite number of repeating fragments complementary to circular DNA. The RCA approach has been widely studied and applied in the biomedical field to areas such as whole genome DNA detection, single nucleotide polymorphism detection, in situ cell detection, and immunochip detection [16]. Ge et al. used the target to initiate RCA and in situ fluorescence detection of microRNAs in tumor cells with high sensitivity and ease of operation [17]. Liu et al. developed a simple isothermal DNA amplification method by combining DNA amplification with molecular recognition to obtain DNA index amplification by RNA-cleaving DNAzyme (RCD) and RCA [18]. Zhou et al. exploited RCA to effect chain displacement and detect microRNAs by monitoring changes in fluorescence [19]. Thus, it can be seen that RCA has become a popular signal amplification method for biological analysis, with wide application, simple operation, and high selectivity.

DNAzymes are single stranded DNA fragments with phosphodiester cleavage activity, structural recognition ability, and high catalytic activity. Some DNAzymes exhibit metal specificity, however, most DNAzymes require metal ions to initiate activity. Kim et al. studied the metal dependence and activity of 8-17 DNAzyme using a fluorescence resonance energy transfer method [20]. Using a self-dividing DNAzyme that is highly selective for mercury ions, Hollenstein et al. developed a highly selective mercury ion sensor that is not stimulated by any other metal cation and has the advantage of being active over a wide concentration range [21]. Wen et al. used a combination of RCA, nicking enzyme signal amplification, and DNAzyme to devise a highly sensitive colorimetric detection method for microRNA affording detection limits as low as 2 amol L^−1^ [22]. The DNAzyme featured specific recognition properties and binds to the RCA, which amplified the detection signal to achieve dual signal amplification. DNAzyme recognized the amplified chain and released the fluorescence by the cleavage of metal ions. The amplified strand may be recycled and continuously combined with DNAzyme to generate enhanced fluorescence.

In the present study, we designed a fluorescence biosensor, which consisted of target-induced rolling-loop amplification with Mg^2+^ recognition and self-cleaving DNAzyme for cyclic amplification of the fluorescence signal of microRNAs. The signal amplification of this sensor is of great significance in the context of ultratrace detection of small biomolecules. The method affords detection limits at the 0.49 pmol L^−1^ level and can provide high sensitivity, specificity, and accuracy for measurement of microRNAs in biological assays.

## 2. Materials and Methods

### 2.1. Reagents and Materials

#### 2.1.1. Materials

The microRNAs, the padlock probe and DNAzyme, were synthesized by Sangon Biotech Co., Ltd. (Shanghai, China). T4 DNA ligase, phi29 DNA polymerase, exonuclease I (EXO I), ribonuclease inhibitor, BSA, (NH4)_2_SO_4_, and Tris-buffer solution (1 mol L^−1^; pH 8.0) were purchased from Sangon Biotech Co., Ltd. (Shanghai, China). dNTP mix (25 mmol L^−1^ each) was purchased from Solarbio. Cell culture-grade ultrapure water was purchased from KeyGEN BioTECH. Magnesium chloride was purchased from Shanghai Macklin Biochemical industry (Shanghai, China). The sequences of nucleic acids employed in this study are given in Table 1 in the Supporting Information. 

#### 2.1.2. Construction of the Circulating Detection Platform

The Tris-HCl buffer (1 mol L^−1^; pH 8.0) was diluted to 30 mmol L^−1^ (pH 8.0) with ultrapure water. The (NH_4_)_2_SO_4_ solution (500 mmol L^−1^) and the Mg^2+^ solution (15 mmol L^−1^) were stored at room temperature.

The microRNAs were diluted to 10 μmol L^−1^ with DEPC (diethyl decarbonate) water. The padlock probe and DNAzyme were diluted to 2 μmol L^−1^ with Tris-HCl buffer (30 mmol L^−1^; pH 8.0) and heated to 90 °C. After 5 min, the solutions were placed in a refrigerator at –20 °C for rapid cooling. Next the following solutions were prepared: (1) 20 mg mL^−1^ BSA, 40 U ml^−1^ ribonuclease inhibitor, 4Ku EXO I and 25mmol L^−1^ dNTPs; (2) T4 DNA ligase and ligase buffer (pH 7.8, 400 mmol L^−1^ Tris-HCl, 100 mmol L^−1^ MgCl_2_, 100mmol L^−1^ DTT, 5 mmol L^−1^ ATP); (3) 10 U μL^−1^ phi29 DNA polymerase; (4) 10 X reaction buffer (330 mmol L^−1^), Tris-acetate (pH 7.9 at 37 °C), 1% (v v^−1^), Tween 20, 660 mmol L^−1^ K-acetate, 10 mmol L-1 DTT, 100 mmol L^−1^ Mg-acetate. 

All the above reagents were stored in a refrigerator at 4 °C.

#### 2.1.3. Instrumentation

Fluorescence measurements were performed with a fluorescence spectrophotometer (Model RF-6000; Shimadzu, Kyoto, Japan). The fluorescence dye used was FAM with excitation and emission wavelengths of 494 nm and 522 nm, respectively. The wavelength range for spectral scanning was 494–560 nm.

### 2.2. Method

#### 2.2.1. Design Strategy for miR-21 Detection

**Figure 1:**
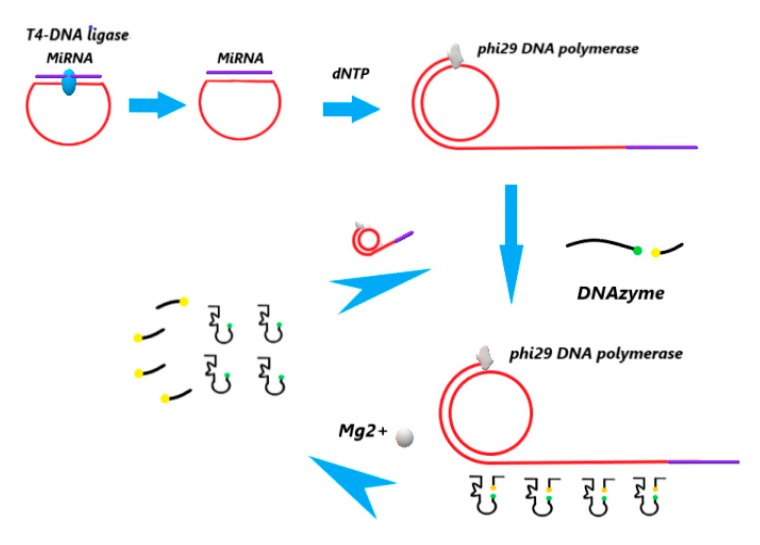
Schematic of microRNA detection based on rolling circle amplification (RCA) and DNAzyme.

As shown in Figure 1, the target microRNA was used as a cyclization template and primer to achieve the RCA reaction. The RCA amplifies the padlock probe fragment to form a long-stranded DNA product with hundreds of repeats, which can be combined with a fluorescently labeled DNAzyme chain to form a target-assisted self-cleaving DNAzyme. Cleavage resulted in a long chain product releasing from the DNAzyme probe, making the dyes apart from the quenchers. After Mg^2+^ cleavage of DNAzyme, the quenching fluorescence of DNAzyme was recovered, and the DNA strands after cleavage were separated from the RCA products. The long chain product can freely combine with the next deoxyribozyme to form the hairpin structure, generate activity, and continue to be cut by Mg^2+^, and thus drives another catalytic cycle.

The two ends of the padlock probe are, specifically, complementary to the target nucleic acid sequence, and are ligated into a loop under the action of T4 DNA ligase; however, when a mismatch occurs, the ligation reaction of the probe cannot be completed. T4 DNA ligase can repair single-stranded incisions in the DNA/RNA complex, connecting the sticky and terminal ends of DNA, and catalyzing the formation of phosphodiester bonds between the adjacent 5’ phosphate groups and the 3’ hydroxyl terminal ends of DNA. Using the cyclized probe as a template, microRNA serves as a primer, and is extended by the phi29 DNA polymerase and dNTPs to generate long-chain DNA of hundreds of tandem repeats. Thus, concentration and amplification of the detection signal are realized. At this point, the free deoxynuclease chain and the RCA products complement each other to form a hairpin structure and produce “activity.” In the presence of Mg^2+^, the substrate fragments on the hairpin structure are cleaved and fluorescence is restored. At the same time, the substrate removes the RCA products and drives another catalytic cycle.

#### 2.2.2. Ligation Reactions

Total of 5 μL of T4 DNA ligase buffer, 1 μL of the padlock probe (25 nmol L^−1^), an appropriate amount of MiR-21 (the dosage of MiR-21 in the optimized test conditions was 25 pmol L^−1^) and 0.5 μL of ribonuclease inhibitor were placed in the sample tube (in the real environment, 5 μL of serum were added into the sample tube). The mixture was incubated at 65 °C for 3 min and held at 37 °C for 30 min. After cooling at room temperature for more than 10 min, 200 U of T4 DNA ligase and 7.5 μL of T4 DNA ligase buffer were added and the solution was held at 37 °C for 2 hours. Finally, 2 μL of Exo I was added and the solution was incubated for 1 hour at 37 °C to eliminate unreacted probes. Exo I is used to digest single-stranded unreacted probes so that the final result is only a ring-locked probe, reducing the error; the solution was then inactivated by holding at 80 °C for 15 min. The probes were joined by ligase to form closed circular DNA. Unused sample may be stored in a refrigerator (4 °C) [23].

#### 2.2.3. The RCA Reactions

Twenty U of phi29 DNA polymerase, 2 μL of phi29 DNA polymerase buffer, 6 μL of dNTP mix, 1 μL of BSA, and 1 μL of (NH_4_)_2_SO_4_ were added to the ligation reaction solution. The RCA reaction was performed for 4 h at 30 °C. The mixture was then held for 5 min at 75 °C to inactivate the polymerase.

#### 2.2.4. Fluorescence Signal Amplification based on DNAzyme

Total of 2 μL of DNAzyme, 12 μL of Mg^2+^, and 6 μL of Tris-HCl buffer (1 mol L^−1^; pH 8.0) were added to the RCA product solution and the mixture was incubated at 37 °C for 2 hours. The final product was stored in a refrigerator (4 °C).

## 3. Results and Discussion

### 3.1. Feasibility Study

**Figure 2:**
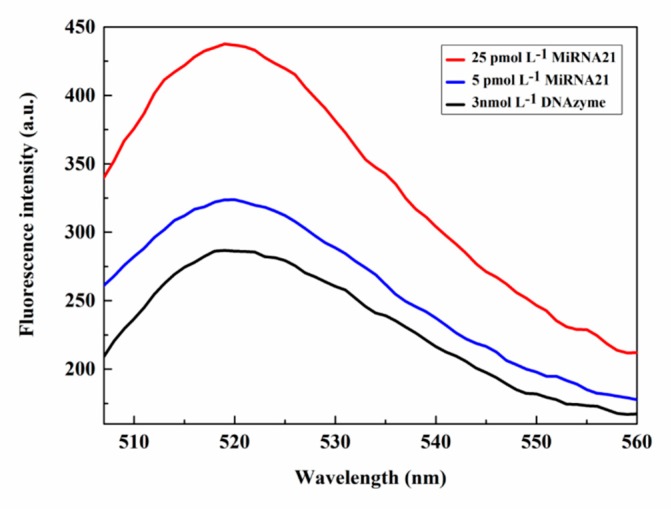
Fluorescence spectra for various concentrations of miR-21.

The feasibility of the experiment was demonstrated for the RCA and DNAzyme reaction by the release of fluorescence, which was initiated by the different concentrations of target miR-21. The spectrum for 5 pmol L^−1^ miR-21 (blue color) in Figure 2 indicates that the fluorescence intensity of the detection platform was slightly higher than the fluorescence intensity of the DNAyme itself (black color) without addition of the target miR-21. The fluorescence spectra corresponding to the different target concentrations, that is, 5 pmol L^−1^, 25 pmol L^−1^, are represented by blue and red colors, respectively. It can be seen that as the concentration increases, the fluorescence intensity released by the detection platform increases progressively. Compared with the DNAzyme detection platform, the fluorescence intensity increases more than 1-fold, indicating the participation of miR-21 in the reaction process, thus confirming the feasibility of the detection platform for RCA amplification and cyclic capture of DNAzyme and cleavage, and release of fluorescence.

### 3.2. Optimization Studies 

#### 3.2.1. Padlock Probe

**Figure 3:**
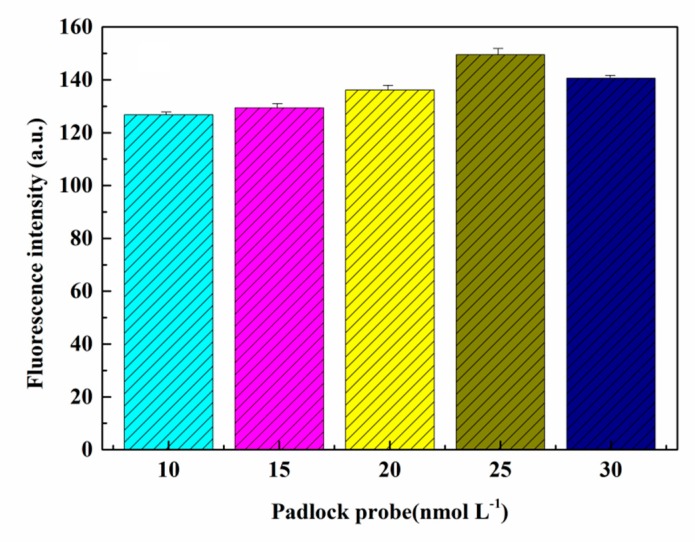
Effect of padlock probe concentration on fluorescence intensity.

For a constant concentration of DNAzyme and fixed conditions for other experimental parameters, the effect on fluorescence of varying the concentration of the padlock probe was investigated. As can be seen from Figure 3, the fluorescence intensity increased gradually with the increase in the padlock probe concentration reaching a maximum intensity at a concentration of 25 nmol L^−1^, beyond which there was a slight decrease in fluorescence signal. Therefore, the concentration of the padlock probe was maintained at 25 nmol L^−1^ in subsequent experiments.

#### 3.2.2. T4 DNA Ligase

**Figure 4:**
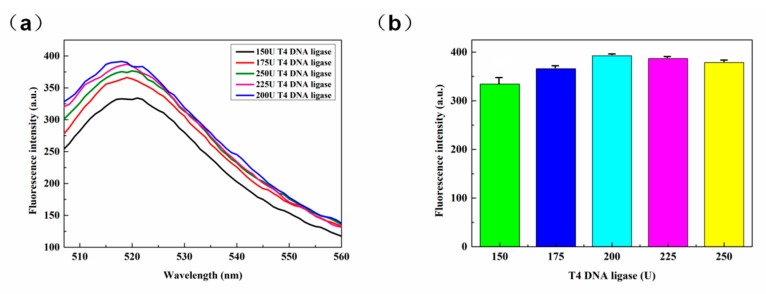
(**a**) Fluorescence spectra for various T4 DNA ligase concentrations; (**b**) comparison of fluorescence intensities for various ligase concentrations.

In the cyclization reaction, the type and content of the ligase is critical, given that the ligase affects the rate and quantity of ring formation. T4 DNA ligase is capable of linking the two adjacent ends and the RNA template complementary to the padlock probe. To optimize the dose, several different detection platforms were constructed. In the absence of ligase, the probe is hydrolyzed by Exo I and cannot form a ring [24]. With regard to Figure 4a and the fluorescence released by different amounts of T4 DNA ligase, maximum fluorescence was realized for 200 U. Further, from inspection of Figure 4b, it can be seen that the fluorescence intensities for 200, 225, and 250 U T4 DNA ligase were similar, indicating that even if the amount of ligase was increased, the amount of cyclization had reached the limit such that fluorescence would not increase. Thus, 200 U was considered to be the optimal amount of ligase for the sensor platform.

#### 3.2.3. Phi29 DNA Polymerase

**Figure 5:**
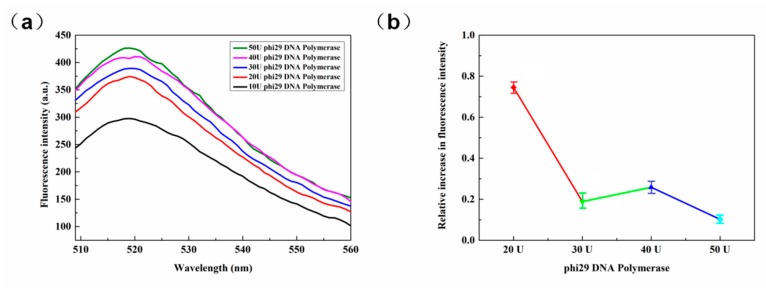
(**a**) Fluorescence spectra for various doses of phi29 DNA polymerase; (**b**) Fluorescence increase percentage comparison chart (521 nm).

Polymerase plays a very important role in the entire RCA reaction and only when the polymerase acts can the entire reaction be initiated. The phi29 DNA polymerase used in the present experiment has high polymerase activity and scalability, and can generate chain replacement reactions by itself without the help of any additional proteins. In the presence of phi29 DNA polymerase and dNTPs, chain extension can be carried out continuously. Conversely, RCA reactions can be difficult to implement. In this experiment, as shown in Figure 5a, it is also evident that the fluorescence intensity increased significantly when the amount of the enzyme increased from 10 U to 20 U. Thereafter, when the amount of enzyme increased, the increase trend of fluorescence intensity decreased. The relative changes in fluorescence are illustrated in Figure 5b, however, when the polymerase was greater than 20 U, the effects on the fluorescence response are not so clear cut. For cost purposes, the amount of polymerase used in subsequent experiments was 20 U.

#### 3.2.4. The Concentration of dNTPs

**Figure 6:**
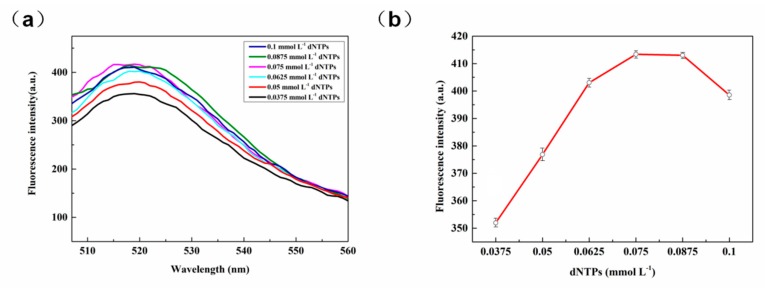
(**a**) Optimization map of dNTPs dose in RCA. (**b**) The effect of various concentrations of dNTPs on fluorescence (521nm).

Deoxyribonucleoside triphosphates (dNTPs) include dCTP, dGTP, dATP, dTTP, which are the basic raw materials required for PCR amplification, fluorescence quantification, reverse transcription, sequencing, and labeling. The dNTPs play a key role in providing energy and the raw materials for the DNA amplification. As can be seen in Figure 6a,b, as the concentration of the dNTPs increased above 0.075 mmol L^−1^, the fluorescence intensity of the system hardly changed. Thus, it can be inferred at this point that the concentration of the dNTPs had little effect on the length of the RCA product, which was governed by the polymerization ability of phi29 DNA polymerase. Accordingly, 0.075 mmol L^−1^ of dNTPs was used in subsequent experiments.

#### 3.2.5. The Concentration of Mg^2+^

**Figure 7:**
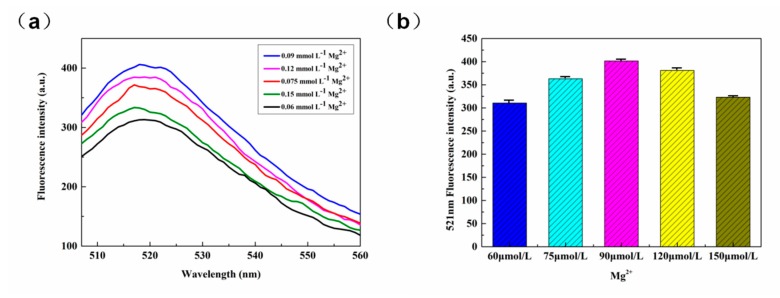
(**a**) Fluorescence spectra for various concentrations of Mg^2+^. (**b**) Fluorescence intensity (521 nm) as a function of Mg^2+^ concentration.

The catalytic activity of DNAzyme is dependent on certain metal ions. In this experiment, we detected the fluorescence of 8-17 DNAzyme under the action of RCA product and Mg^2+^ cleavage. Figure 7a shows that as the Mg^2+^ concentration was increased, the fluorescence intensity underwent stepwise increases, but further increases in concentration beyond 0.09 mmol L^−1^ resulted in a decrease in fluorescence intensities. This trend in the fluorescence response can be more clearly discerned in Figure 7b, where the fluorescence intensity reached a maximum at Mg^2+^ concentration of 0.09 mmol L^−1^.

#### 3.2.6. miR-21 Detection

**Figure 8:**
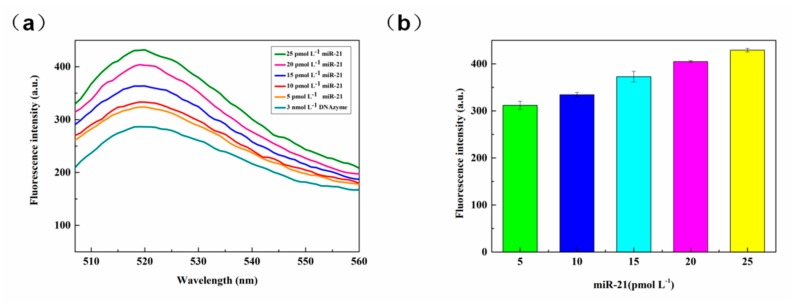
(**a**) Fluorescence spectra for various concentrations of miR-21. (**b**) Plot for the dependence of fluorescence intensity on miR-21 concentration. Error bars indicate five consecutive measurements performed at each concentration (n = 5).

The miR-21 platform was constructed by using the optimized set of experimental conditions as outlined above and using prepared samples of different concentrations for the RCA reaction. The results obtained are shown in Figure 8a. The fluorescence of the DNAzyme itself gave the lowest fluorescence intensity of the detection platform. The addition of miR-21 initiates a rolling circle amplification reaction to increase the fluorescence of the detection platform. On addition of 5 pmol L^−1^ miR-21, the fluorescence of the system did increased. When the concentration of miR-21 was increased to 25 pmol L^−1^, a very high fluorescence response was detected, indicating that the target of this concentration was highly utilized in the system and released a large amount of fluorescence. As the concentration of miR-21 was gradually increased from 5 pmol L^−1^ to 25 pmol L^−1^, the fluorescence intensity of the system increased in a regular manner. After several parallel experiments (n = 5), error analysis was carried out on the obtained data, as shown in Figure 8b. In the figure, the standard deviation of the detected values of each concentration is less than 0.03. It indicates that the repeatability of the sensor is acceptable.

**Figure 9:**
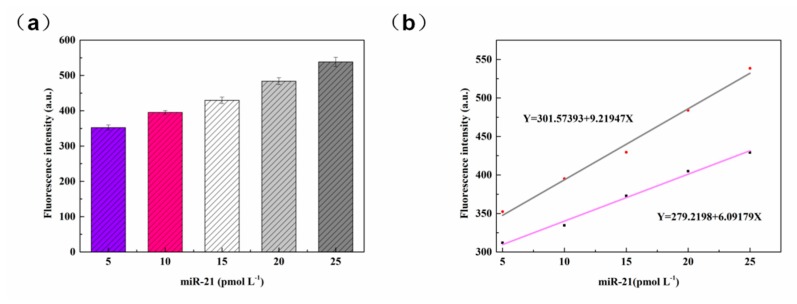
(**a**) Fluorescence spectra of miR-21 at different concentrations in serum. (**b**) The standard curve of miR-21.

In the real environment, samples were tested five times, and the results obtained are shown in Figure 9a. The overall fluorescence intensity was higher than that in Figure 8b. The fluorescence increases regularly in Figure 9a, which is the same as the trend of detecting targets in the buffer environment. The standard curve is obtained according to the detection data, as shown in Figure 9b, pink curve equation Y = 279.2198 + 6.09179 X (where Y = F_(MicroRNA concentration)_) and the detection limit can be as low as 0.49 pmol L^−^^1^ (according to the LOD = 3σ/ S = 0.49 pmol L^−^^1^), which had a correlation coefficient close to 1 (Pearson’s r = 0.99664, r^2^ = 0.99106). The black curve equation Y = 301.57393 + 9.21947 X is the standard curve of the real sample in the serum environment (Pearson’s r = 0.99588, r^2^ = 0.98904). It can be seen that the fluorescence of serum samples is slightly higher than that of the detection system, showing a linear trend. A standard curve is formed between the concentration of 5 pmol L^−^^1^ and 25 pmol L^−^^1^, indicating that the platform has a strong linear relationship at the concentration level of pmol L^−^^1^. The results demonstrated that the platform provided a feasible method for signal amplification and high sensitivity detection of microRNAs. Compared with most other literatures (Table 2), the linearity of the sensor is better and the detection limit can reach pM. Therefore, we can conclude that this method is very suitable for the detection of microRNAs in this linear range, providing a feasible scheme for sensors to detect small molecules.

#### 3.2.7. Selectivity of microRNA Detection

**Figure 10:**
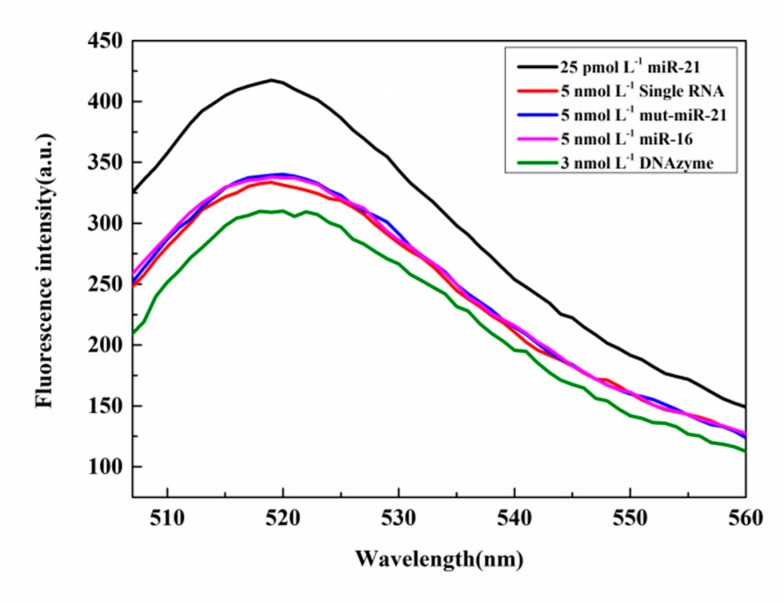
Specific detection of miR-21.

To test for selectivity, different primers were used to initiate the RCA reaction and to evaluate the performance of the sensor. Under the same conditions, mut-miR-21, single RNA, miR-16, and miR-21 were subjected to the RCA reaction, respectively. Figure 10 shows that because of the multiple signal amplification, the fluorescence intensity of the miR-21 sample was significantly enhanced even at the pmol L^−1^ level of the target primer. The fluorescence intensities for the other three primer samples (5 nmol L^−1^ mut-miR-21, 5 nmol L^−1^ single RNA, and 5 nmol L^−1^ miR-16) were much lower and differed little from the fluorescence intensity of the DNAzyme itself. These results confirm that this sensor system is highly sensitive, selective, and specific in detecting microRNA.

## 4. Conclusions

A highly sensitive and selective microRNA sensor exploiting target-induced RCA and self-cleaving DNAzyme has been developed. The sensor exhibited fast response, the detection limit can be as low as 0.49 pmol L^−^^1^, and the sensor platform can design target detection platform of different concentration levels by changing the concentration of experimental conditions. The biosensor holds promise as a universal platform for the analysis of microRNAs with potential for new applications in disease prevention and treatment and drug discovery.

## Figures and Tables

**Table 1:** DNA sequences.

Name	Sequence(5’–3’)
Padlock probe	pCTGATAAGCTATTTATTTCCTCAATGCTGCTGCTGTACTACTAGTGATTTACTTGGATGTCTTCAACATCAGT
DNAzyme	TTTATTTCAAACT(Dabcyl)rAGGT(FAM)CTTTTTTTTTGACTCCGAGCCGGACGAAGTTAATGCTG
MiR-21	UAGCUUAUCAGACUGAUGUUGA
MiR-16	UAGCAGCACGUAAAUAUUGGCG
mut-miR-21	UAGCUUA**A**CAGACUGAUGUUGA
Single RNA	UUGUACUACACAAAAGUACUG

**Table 2:** Comparison of other methods for microRNAs detection.

Methods	LOD	Linear Range	Correlation Coefficient (R^2^)	Reference
DNA self-assembled molecular tweezers	0.6 pM	1–500 nM	0.9929	[25]
Graphene oxide for rapid microRNA detection	/	50–400 nM	0.9562	[26]
Fluorescence quenching of graphene oxide integrating	3.0fM	0.02–100 pM	/	[27]
A novel DNA nanomachine based on the linear rolling circle amplification strategy	87fM	0.1 pM–0.1 nM	0.9908	[13]
Highly sensitive determination of microRNA using target-primed and branched rolling-circle amplification	0.25pM	0.025 pM–2.5 nM	0.9994	[23]
Fluorometric determination of microRNA based on strand displacement amplification and rolling circle amplification	1.04fM	10 fM–0.1 nM	0.9957	[19]
Cascade amplification by catalytic DNAzymes	10pM	10 pM–100 nM	/	[28]
Fluorescence quenching of gold nanoparticles with a competitive hybridization	33.4fM	100 fM–1.0 nM	/	[29]
DNAzyme-based target-triggered rolling-circle amplification	0.49pM	0–25 pM	0.99106	This work
